# MiR-122 overexpression alleviates oxygen–glucose deprivation-induced neuronal injury by targeting sPLA2-IIA

**DOI:** 10.3389/fneur.2024.1395833

**Published:** 2024-05-10

**Authors:** Yuanfang Yu, Pan Li, Mengyuan Chen, Wenfeng Zhan, Ting Zhu, Ling Min, Hao Liu, Bo Lv

**Affiliations:** ^1^Guangdong Cardiovascular Institute, Guangdong Provincial People’s Hospital, Guangdong Academy of Medical Sciences, Guangzhou, China; ^2^Department of General Practice, Guangdong Geriatrics Institute, Guangdong Provincial People's Hospital (Guangdong Academy of Medical Sciences), Southern Medical University, Guangzhou, China; ^3^Affiliated Cancer Hospital and Institute of Guangzhou Medical University, Guangzhou, China; ^4^Department of Laboratory Medicine, Affiliated Cancer Hospital and Institute of Guangzhou Medical University, Guangzhou, China

**Keywords:** microRNA-122-5p, ischemic stroke, astrocytes, cytokines, sPLA2-IIA

## Abstract

**Background:**

Ischemic stroke (IS) is a neurological disease with significant disability and mortality. MicroRNAs were proven to be associated with cerebral ischemia. Previous studies have demonstrated miR-122 downregulation in both animal models of IS and the blood of IS patients. Nonetheless, the role and mechanism of miR-122-5p in IS remain unclear.

**Methods:**

We established primary human and mouse astrocytes, along with HT22 mouse hippocampal neuronal cells, through oxygen–glucose deprivation/reoxygenation (OGD/R) treatment. To assess the impact of miR-122, we employed CCK8 assays, flow cytometry, RT-qPCR, western blotting, and ELISA to evaluate cell viability, apoptosis, reactive oxygen species (ROS) generation, and cytokine expression. A dual-luciferase reporter gene assay was employed to investigate the interaction between miR-122 and sPLA2-IIA.

**Results:**

Overexpression of miR-122 resulted in decreased apoptosis, reduced cleaved caspase-3 expression, and increased cell viability in astrocytes and HT22 cells subjected to OGD/R. RT-qPCR and ELISA analyses demonstrated a decrease in mRNA and cytokine levels of interleukin (IL)-6 and tumor necrosis factor (TNF)-α in both astrocytes and HT22 cells following miR-122 overexpression. Moreover, miR-122 overexpression reversed OGD/R-induced ROS levels and 8-OHdG formation in astrocytes. Additionally, miR-122 overexpression decreased the mRNA and protein expression of inducible nitric oxide synthase (iNOS). Furthermore, we found that miR-122 attaches to the 3′-UTR of sPLA2-IIA, thereby downregulate its expression.

**Conclusion:**

Our study demonstrates that miR-122-mediated inhibition of sPLA2-IIA attenuates OGD/R-induced neuronal injury by suppressing apoptosis, alleviating post-ischemic inflammation, and reducing ROS production. Thus, the miR-122/sPLA2-IIA axis may represent a promising target for IS treatment.

## Introduction

Stroke, characterized by its significant morbidity and disability burden, is one of the most devastating cerebrovascular diseases and the third leading cause of death worldwide, posing a significant public health concern. IS is an episode of neurological dysfunction caused by focal cerebral infarction resulting from arterial stenosis or occlusion. It represents for approximately 85% of all stroke cases (1). Despite significant advancements in treatment, devising an effective therapeutic strategy for mitigating ischemic brain damage remains a formidable challenge. A substantial induction of pro-inflammatory cytokines and chemokines (including IL-6, IL-1β, TNF-α, IFN-γ, CXCL1, MCP-1) were released within minutes after IS, which is a significant contributor to neuronal loss (2–5). Astrocytes, the most abundant cells in the central nervous system and crucial components of the blood–brain barrier (6). Induced by inflammatory factors (such as IL-1, TNF-α) and chemokines like CCL2 released by microglia, astrocytes polarize into A1 reactive astrocytes characterized by C3/iNOS expression (7). Current understanding suggests that A1 reactive astrocytes exacerbate neuroinflammation by further releasing TNF-α, IL-6, while A2 reactive astrocytes exert neuroprotective effects by releasing neurotrophic factors and facilitating glutamate reuptake, thus playing a role in mitigating cytotoxicity (8, 9). Previous study have demonstrated a significant increase in IL-6, TNF-α, and iNOS expression in astrocytes following OGD, with iNOS(+) reactive astrocytes leading to increased neuronal apoptosis (10), suggesting the involvement of A1 astrocytes in post-IS inflammation and oxidative stress. Moreover, it has been found that edaravone inhibits the transformation of A1 astrocytes by suppressing the expression of inflammatory cytokines and chemokines (11). Additionally, astrocytes play a critical role in the redox homeostasis during ischemic stroke, as evidenced by the significantly higher glutathione (GSH) content in astrocytes compared to neurons during OGD, with mitochondrial transfer from astrocytes to neurons aiding in maintaining neuronal mitochondrial function (12). On the other hand, activated astrocytes further promote microglial activation and induce apoptosis of damaged cells by producing IL-1β (13). In summary, astrocytes play important roles in inflammation, oxidative stress, and cell apoptosis in ischemic stroke.

MicroRNAs (miRNAs) comprise a group of small RNAs that are highly conserved, endogenous, non-coding and widely distributed across eukaryotes. They function primarily in the post-transcriptional regulation of gene expression (14, 15). An expanding body of evidence underscores that miRNAs play a crucial role in the pathophysiology of IS. These miRNAs exert their influence by exaggerating inflammatory responses, impairing the BBB, and inducing cytotoxicity and apoptosis, alongside contributing to vascular injury and facilitating regeneration processes. IS, which arises from ischemia and hypoxia, may trigger alterations in miRNA expression levels (16). These alterations are implicated in the progression of IS as they govern the expression of cytokines and immunomodulatory factors. Previous studies have provided evidence indicating a significant decrease in the levels of miR-122 in both rat models of middle cerebral artery occlusion and reperfusion (MCAO/R), as well as the blood of patients with acute stroke (17–19). Our previous study revealed that treatment with miR-122-5p mimics reduces the volume of cerebral infarction in rats. Furthermore, we validated that miR-122-5p reduces iNOS expression in leukocytes and brain microvascular endothelial cells (BMVECs), suggesting a cerebroprotective role of miR-122 in IR injury following IS (20, 21). Nevertheless, the role and mechanism of miR-122-5p in IS remain unclear.

Phospholipase A2 (PLA2) superfamily is a group of enzymes that hydrolyze the ester bond at the second position on the glycerol backbone of phospholipid molecules (22). Previous studies have revealed the presence of PLA2s in the cerebral cortex following ischemia (23). PLA2s act as rate-limiting factors in the production of bioactive substances, such as arachidonic acid (AA), prostaglandins, and platelet-activating factor (PAF). Therefore, both the concentration and activity of PLA2s are considered independent biomarkers of IS (23). Utilizing predictive analysis software such as TargetScan and miRanda, we suggest that miR-122-5p may exert a regulatory effect on the target gene, secreted PLA2 group IIA (sPLA2-IIA) (20). Therefore, the aim of this study was to examine the regulatory influence of miR-122 on sPLA2-IIA expression and elucidate the protective effects and underlying mechanism of miR-122 in astrocytes subjected to OGD/R-induced injury.

## Materials and methods

### Cell culture

This study was approved by the Ethics Committee of Guangdong Provincial People’s Hospital (Grant No. KY2020-563-01). Primary human astrocytes (CP-H122), mouse astrocytes (CP-M157), mouse hippocampal neuronal cell line (HT22, CL-0697) were all obtained from Wuhan Procell Life Science and Technology Co., Ltd. (Wuhan, China). The cells were all cultured in Dulbecco’s Modified Eagle Medium (DMEM) supplemented with 10% fetal bovine serum (FBS) and a 1% mixture of penicillin and streptomycin. All cell cultures were maintained in an incubator with constant temperature and carbon dioxide levels, specifically at 37°C and 5% CO_2_.

### Cell transfection

The miR-122-5p mimic and its corresponding negative control (NC) were chemically synthesized by Guangzhou RiboBio Co., Ltd. (Guangzhou, China), following established protocols. The miR-122-5p mimic and its NC were transfected into the cells using Lipofectamine 3,000 (Invitrogen, Thermo Fisher Scientific Inc.) according to the manufacturer’s instructions.

### OGD/R cell model

The vitro cerebral I/R injury was simulate utilizing OGD/R-treated cells. To induce OGD stimulation, the cells were cultured in hypoxic incubator with 5% CO_2_, 1% O_2_, and 94% N2 for 12 h, using DMEM medium devoid of glucose and serum. Following OGD stimulation, the cells were subsequently incubated under normoxic conditions (5% CO2 and 95% air) for 24 h using DMEM medium supplemented with 10% FBS and glucose to simulate reperfusion. The control group was defined as cells cultured in normal DMEM medium under normoxic conditions in an incubator.

### CCK-8 assays

According to the manufacturer’s instructions for the Counting Kit-8 assay (APExBio Technology), cell viability measurements were performed by seeding 2 × 10^4^ cells in 100 μL of complete culture media mixed with 10 μL CCK8 reagent per well in 96-well plates, after transfection with miR-122 or its NC for the specified duration.

### Cell apoptosis assay

Perform cell apoptosis detection according to the instructions provided in the Annexin V-FITC/PI Cell Apoptosis Detection Kit (TransGen Biotech, Beijing, China). Collect the treated cells from each group as instructed, followed by a thorough washing step utilizing PBS. Subsequently, resuspended the cells in the Annexin V Binding buffer, then proceed to stain the cells with Annexin V-FITC and PI and incubate the mixture in a light-protected environment for 15 min at room temperature. Next, combine the cell suspension with Annexin V Binding buffer, place it on ice, and finally, determine the apoptosis rate by flow cytometry within 1 h.

### Real-time quantitative RT-PCR

Total RNA was extracted using the E.Z.N.A. HP Total RNA kit (Omega Bio-tek, United States). The cDNA synthesis was carried out using 0.5 μg of RNA with the Prime Script RT master mix (Perfect Real Time; TaKaRa, Japan). Quantitative real-time PCR analysis was conducted in triplicate on LightCycler 480 (Roche, Mannheim, Germany) using SYBR Premix Ex Taq (TaKaRa, Japan) and the datas were normalized based on the expression of GAPDH RNA. The results were calculated utilizing the ΔΔCT methods. The primers for selected genes were as follows:

h-iNOS: 5′-AGCTGAACTTGAGCGAGGAG-3′, 5′-GGAAAAGACTGCACCGAAGA-3′;

h-TNF-α: 5′-GTGCTTGTTCCTCAGCCTCTT-3′, 5′-ATGGGCTACAGGCTTGTCACT-3′;

h-IL-10: 5′-ACCTGCCTAACATGCTTCGAG-3′, 5′-CTGGGTCTTGGTTCTCAGCTT-3′;

h-sPLA2-IIA: 5′-TGACGACAGGAAAGGAAGCC-3′, 5′-CTGCTCCCCGAGTTGCTAAA-3′;

h-GAPDH: 5′-GCACCGTCAAGGCTGAGAAC-3′, 5′-TGGTGAAGACGCCAGTGGA-3′;

m-IL-6: 5′-CCAAGCCTTATCGGAAATGA-3′, 5′-TTTTCACAGGGGAGAAATCG-3′;

m-TNF-α: 5′-CGGTGCCTATGTCTCAGCCT-3′, 5′-GAGGGTCTGGGCCATAGAAC-3′;

m-sPLA2-IIA: 5′-CTGTTGCTACAAGAGCCTGG-3′, 5′-GCCGTTTCTGACAGGAGTTC-3′;

m-GAPDH: 5′-TGTGTCCGTCGTGGATCTGA-3′, 5′- TTGCTGTTGAAGTCGCAGGAG-3′.

### Western blotting

Cell lysis was performed using Radioimmunoprecipitation assay buffer (Beyotime Biotechnology, China) to disrupt cellular membranes and release intracellular components. The concentration of total protein was detected using the BCA Protein Assay Kit (Thermo Fisher, Waltham, MA, United States). The proteins were then separated by 8–12% Sodium dodecyl sulfate-polyacrylamide gel electrophoresis using an electric current, and subsequently transferring them to polyvinylidene fluoride (PVDF) membranes. The PVDF membranes were blocked with 5% skim milk, and then incubated with primary antibodies overnight at 4°C. Following primary antibody incubation, the membranes were washed to remove unbound antibodies and then incubated with the secondary antibodies for an hour at room temperature. Bound antibodies were visualized by ECL reagents (Thermo Fisher). In this study, the primary antibodies consist of anti-β-Actin antibody (#3700, Cell Signaling Technology), anti-iNOS (#68186, Cell Signaling Technology), sPLA2-IIA (sc-58363, Santa Cruz Biotechnology), anti-caspase-3 antibody (#9661, Cell Signaling Technology).

### Measurement of cytokine production

The supernatants were collected after the aforementioned steps and stored at −80°C. The levels of TNF-α and IL-10 were measured utilizing sandwich ELISA with an ELISA kit (eBioscience, San Diego, CA, United States), according to the manufacturer’s protocols.

### Measurement of intracellular ROS

The ROS expression were detected utilizing the fluorescent probe 2,7-dichlorodihydrofluorescein diacetate (DCFH-DA; Beyotime Institute of Biotechnology, Beijing, China) following the manufacturer’s protocols. Intracellular ROS could oxidize non-fluorescent DCFH, the hydrolysis product of DCFH-DA upon cellular entry, to green fluorescent 2,7-dichlorofluorescein, with fluorescence intensity directly proportional to the cellular ROS levels. Subsequently, the treated cells were incubated with DCFH-DA at a temperature of 37°C for 20 min, and then the fluorescence was detected using flow cytometry.

### Measurement of 8-hydroxy-2′-deoxyguanosine

The oxidative stress-associated marker 8-OHdG was assessed utilizing the OxiSelect™ Oxidative DNA Damage ELISA Kit (8-OHdG Quantitation, Trial Size) (Cell Biolabs Inc., United States). After the indicated treatments, cell supernatants were collected, and the 8-OHdG levels were quantified following the manufacturer’s protocol.

### Luciferase reporter assay

sPLA2-IIA containing the predicted miR-122-5p binding site were cloned into pGL3-sPLA2-IIA-Wt (wild-type) and pGL3-sPLA2-IIA-Mut (mutant type) (RiboBio Co., Ltd. Guangzhou, China), respectively. The Wt or Mut 3′-UTR of sPLA2-IIA vector and miR-122-5p mimic or its NC were co-transfected into 293 T cells utilizing Lipofectamine 3000 (Invitrogen, Thermo Fisher Scientific Inc.). The luciferase activity within the cells was measured 48 h post-transfection utilizing the luciferase assay system (Ambion, Austin, TX, United States).

### Statistical analysis

Results were expressed as mean ± SD based on three independent experiments unless except where noted differently. Formal analysis between two groups were performed using a two-tailed unpaired Student’s *t*-test, with SD denoted by bars. All statistical analyses were conducted using GraphPad Prism 5.0 (GraphPad Software Inc., La Jolla, CA, United States). A *p*-value of <0.05 was considered to be statistically significant.

## Results

### MiR-122 decreases astrocyte apoptosis following an IS *in vitro*

**Figure 1:**
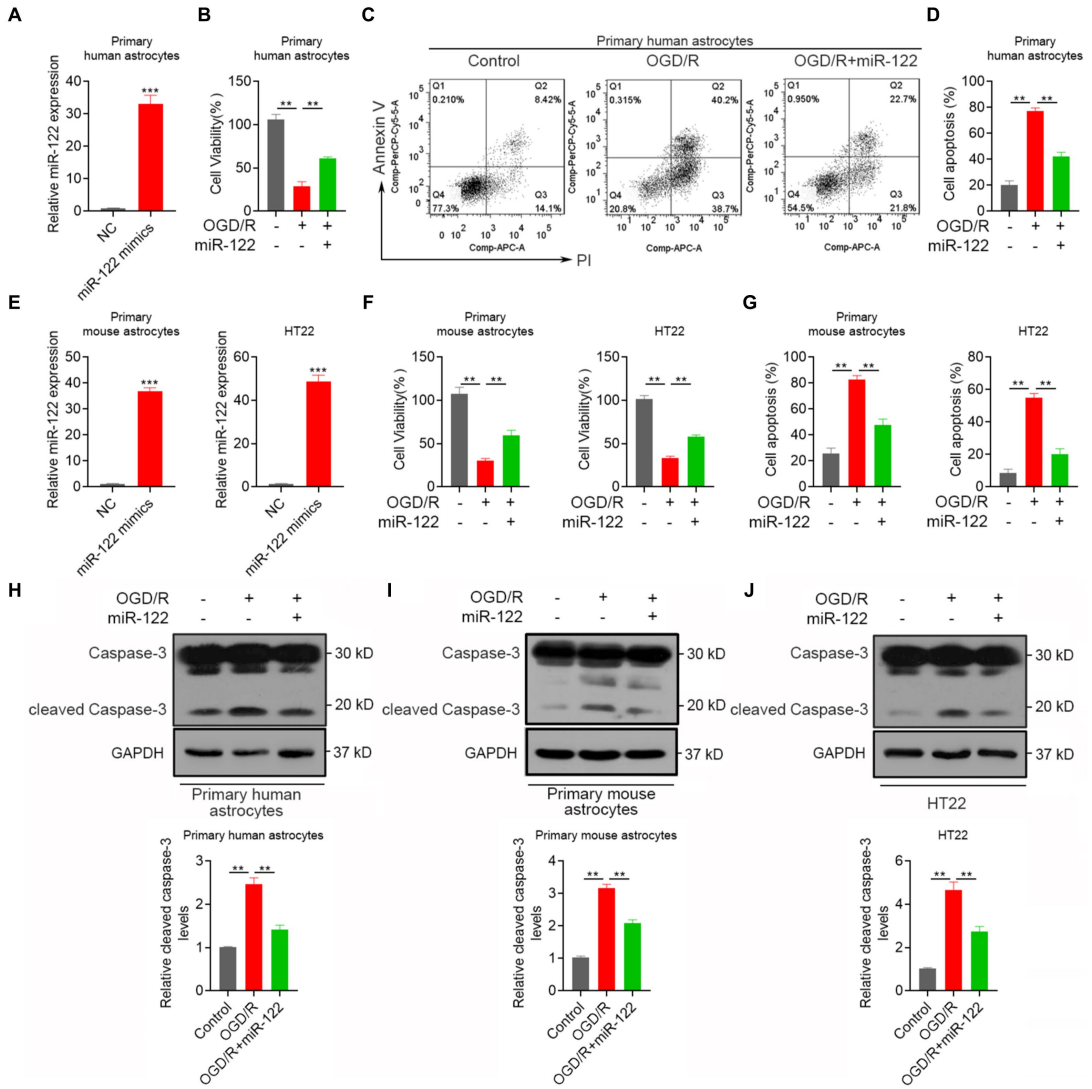
MiR-122 decreases astrocyte apoptosis after IS *in vitro*. **(A)** Primary human astrocytes were transfected with miR-122 mimics and NC, the expression of miR-122 was measured by qRT-PCR. **(B)** Primary human astrocytes transfected with miR-122 mimics and NC were treated with OGD, cell viability was measured by CCK8 assay. **(C,D)** Primary human astrocytes transfected with miR-122 mimics and NC were treated with OGD, cells were then stained with Annexin V-APC and propidium iodide, cell apoptosis was analyzed by flow cytometry. **(E)** Primary mouse astrocytes and HT22 mouse hippocampal neuronal cells were transfected with miR-122 mimics and NC, the expression of miR-122 was measured by qRT-PCR. **(F,G)** Primary mouse astrocytes and HT22 mouse hippocampal neuronal cells transfected with miR-122 mimics and NC were treated with OGD, **(F)** cell viability was measured by CCK8 assay, **(G)** cell apoptosis was analyzed by flow cytometry. **(H)** Primary human astrocytes transfected with miR-122 mimics and NC were treated with OGD, the expression of cleaved caspase-3 were by western blot. **(I)** Primary mouse astrocytes transfected with miR-122 mimics and NC were treated with OGD, the expression of cleaved caspase-3 were by western blot. **(J)** HT22 mouse hippocampal neuronal cells transfected with miR-122 mimics and NC were treated with OGD, the expression of cleaved caspase-3 were by western blot. Each point represents the mean ± SD. Data show a representative of three independent experiments. **p < 0.01, ***p < 0.001.

Astrocytes play a pivotal role in maintaining brain homeostasis, with their activation marking an early response to ischemia–reperfusion IR injury. Apoptosis serves as a major mechanism contributing to neuronal loss after an IS. We therefore explored the impact of miR-122 on the apoptosis response of astrocyte *in vitro*. RT-qPCR analysis demonstrated that miR-122 mimic transfection successfully resulted in significant overexpression of miR-122 in astrocytes (Figure 1A). Importantly, treatment with miR-122 mimics promoted the survival of primary human astrocytes subjected to OGD/R (Figure 1B). Concurrently, miR-122 mimics significantly decreased apoptosis among astrocytes cultured under OGD/R conditions (Figures 1C,D), indicating that miR-122 might be implicated in astrocyte apoptosis triggered by ischemic conditions. Similar results were observed with primary mouse astrocytes and HT22 mouse hippocampal neuronal cells treated with miR-122 mimics (Figures 1E–G). Moreover, we evaluated the expression of cleaved caspase-3, an apoptosis indicator, in primary astrocytes and HT22 cells through western blot analysis. In this study, we found that treatment with miR-122 mimics markedly reversed the expression of cleaved caspase-3 induced by OGD/R in both primary astrocytes and HT22 cells (Figures 1H–J).

### MiR-122 inhibits pro-inflammatory

**Figure 2:**
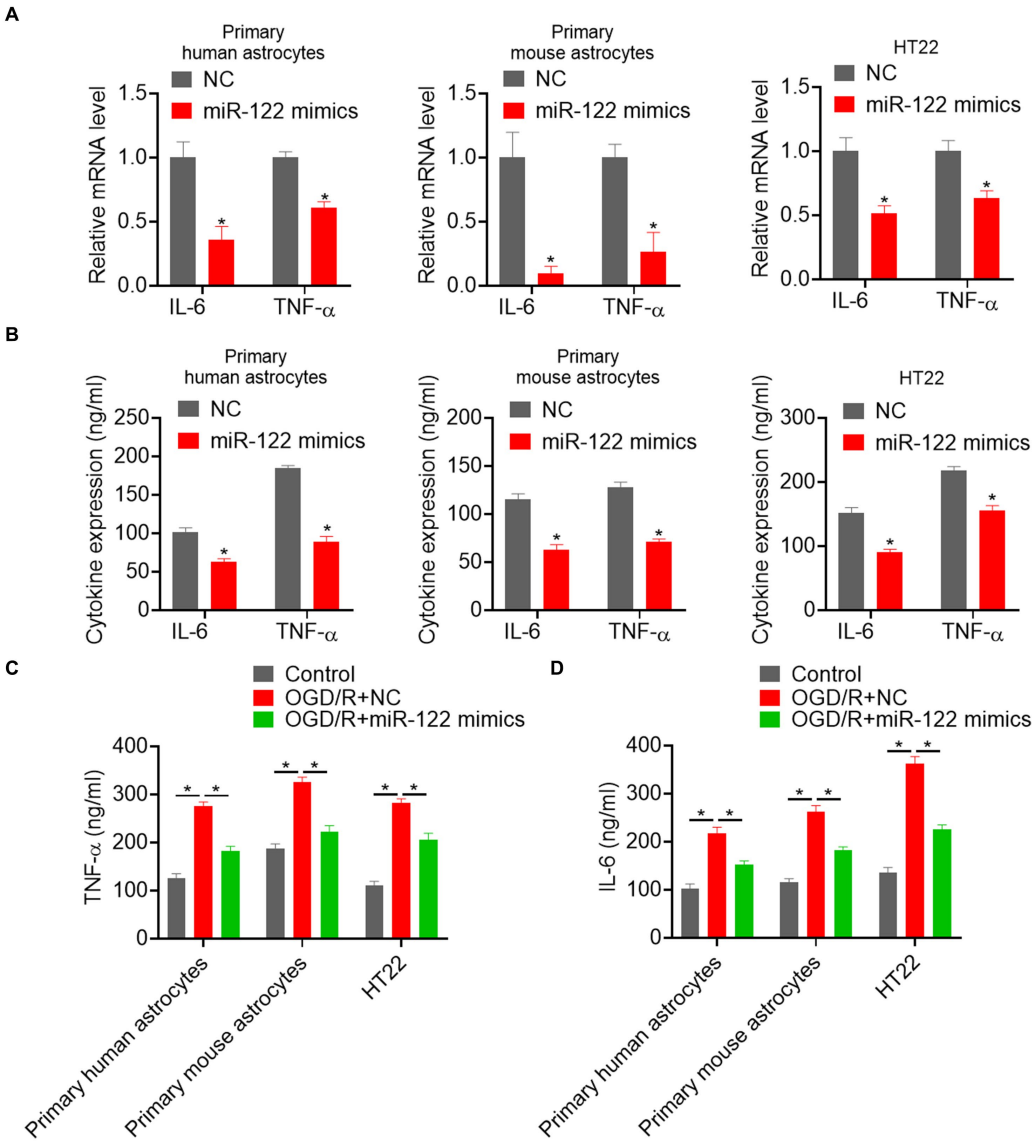
MiR-122 inhibits pro-inflammatory responses. **(A)** Primary human and mouse astrocytes, and HT22 mouse hippocampal neuronal cells were transfected with miR-122 mimics and NC, the expression of IL-6 and TNF-α were measured by qRT-PCR. **(B)** Primary human and mouse astrocytes, and HT22 mouse hippocampal neuronal cells were transfected with miR-122 mimics and NC, the expression of IL-6 and TNF-α were measured by ELISA. **(C)** Primary human and mouse astrocytes, and HT22 mouse hippocampal neuronal cells transfected with miR-122 mimics and NC were treated with OGD, the expression of TNF-α were measured by ELISA. **(D)** Primary human and mouse astrocytes, and HT22 mouse hippocampal neuronal cells transfected with miR-122 mimics and NC were treated with OGD, the expression of IL-6 was measured by ELISA. Each point represents the mean ± SD. Data show a representative of three independent experiments. *p < 0.05, **p < 0.01.

Previous studies have suggested that strokes can trigger an inflammatory response that promotes astrocyte activation. Thus, we evaluated the effects of miR-122 on the expression of pro-inflammatory factor. We found a significant reduction in the mRNA expression of the IL-6 and TNF-α in astrocytes and HT22 cells overexpressing miR-122 (Figure 2A). ELISA showed a significant reduction in IL-6 and TNF-α following miR-122 treatment in both astrocytes and HT22 cells (Figure 2B). We further examined the impact of miR-122 on pro-inflammatory effects induced by OGD/R. The results showed that OGD/R resulted in a significant increase in the expression of IL-6 and TNF-α, whereas treatment with miR-122 mimics significantly reduced IL-6 and TNF-α expression in both astrocytes and HT22 cells (Figures 2C,D).

### MiR-122 inhibits oxidative responses

**Figure 3:**
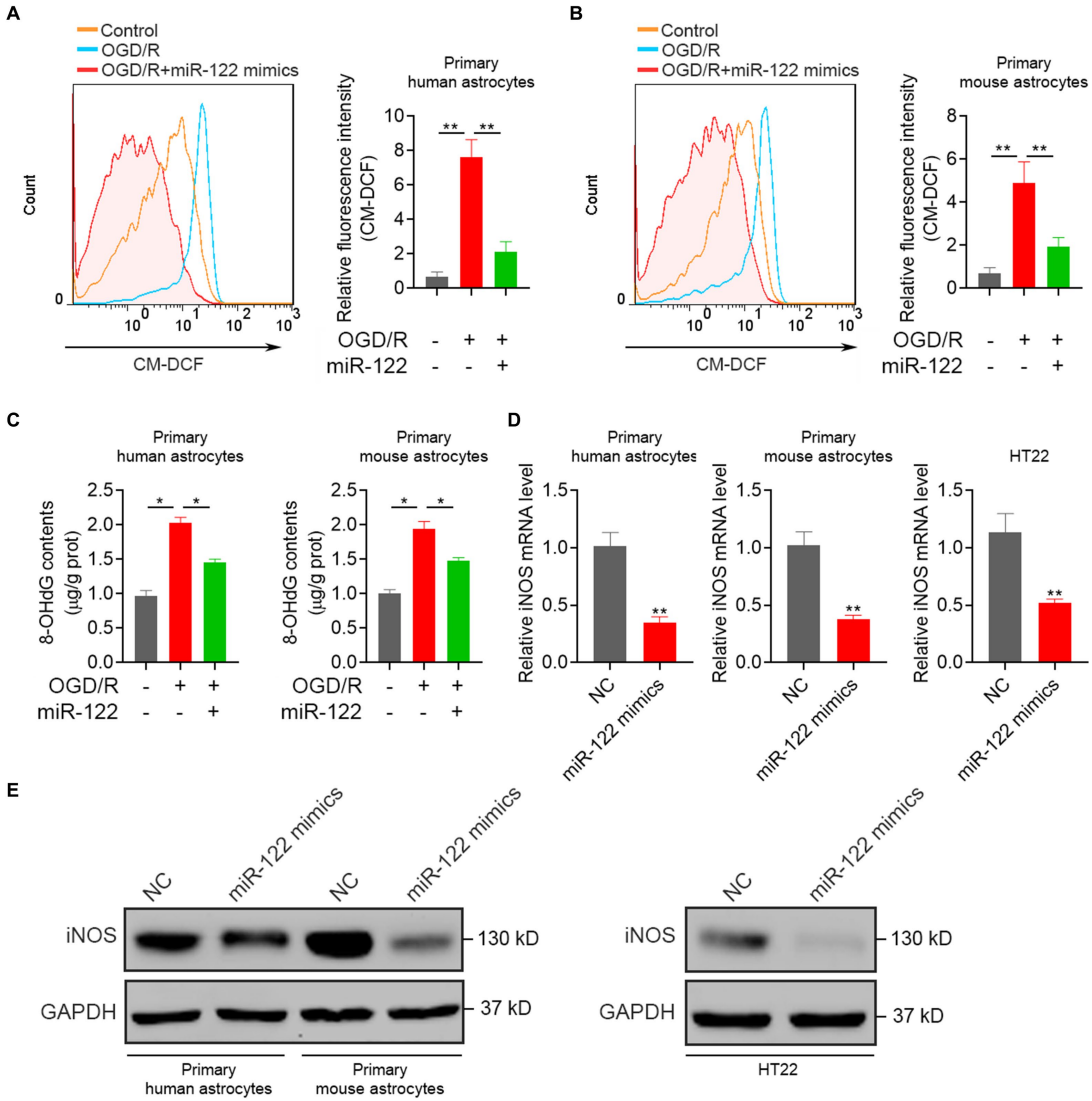
MiR-122 inhibits oxidative responses. **(A)** Primary human astrocytes transfected with miR-122 mimics and NC were treated with OGD, the levels of ROS were analyzed by flow cytometry. **(B)** Primary mouse astrocytes transfected with miR-122 mimics and NC were treated with OGD, the levels of ROS were analyzed by flow cytometry. **(C)** Primary human and mouse astrocytes transfected with miR-122 mimics and NC were treated with OGD, 8-OHdG contents were measured. **(D,E)** Primary human and mouse astrocytes, and HT22 mouse hippocampal neuronal cells were transfected with miR-122 mimics and NC, **(D)** the expression of iNOS were measured by qRT-PCR, **(E)** the expression of iNOS were measured by western blot. Each point represents the mean ± SD. Data show a representative of three independent experiments. *p < 0.05, **p < 0.01.

ROS production serves as an initial trigger for ischemic brain injury. Consequently, this study examined the impact of miR-122 on oxidative damage induced by OGD/R in astrocytes. We found that OGD/R resulted in a significant upsurge in ROS production, whereas treatment with miR-122 mimics significantly reduced ROS production in both primary human and mouse astrocytes (Figures 3A,B). Moreover, treatment with miR-122 mimics significantly attenuated the generation of 8-OHdG, thereby attenuating oxidative stress injury (Figure 3C). Furthermore, we also assessed the impact of miR-122 mimics on iNOS expression levels, a critical enzyme involved in the synthesis of ROS and nitric oxide. We observed a significant attenuation of both mRNA and protein expression of iNOS upon miR-122 overexpression in astrocytes and HT22 cells (Figures 3D,E). These results indicate that miR-122 diminishes ROS production, alleviates oxidative stress, and consequently inhibits astrocyte apoptosis in response to the OGD/R challenge.

### sPLA2-IIA is a direct target of miR-122

**Figure 4:**
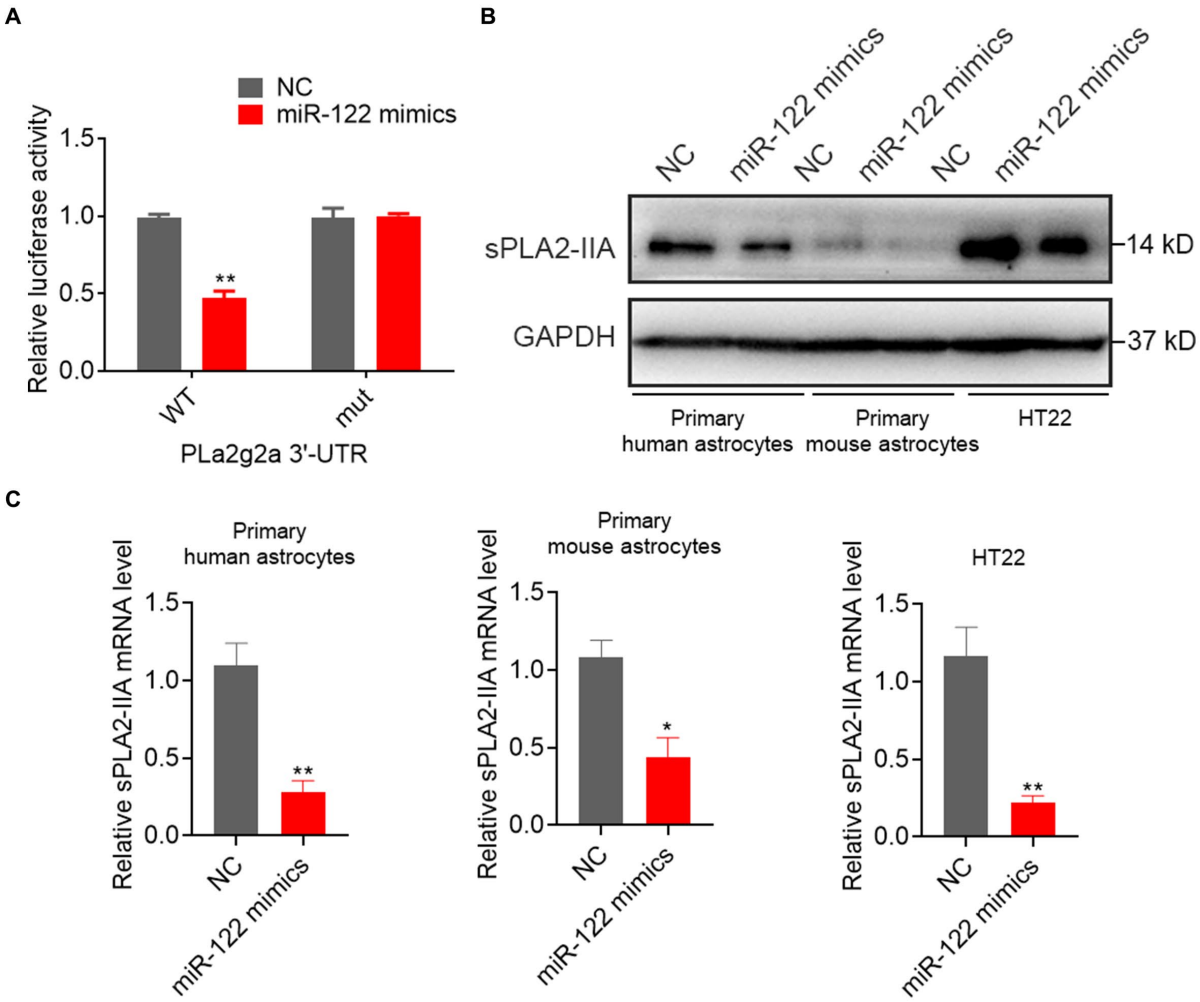
miR-122 directly targeted sPLA2-IIA 3′UTR to suppress its expression. **(A)** HT22 mouse hippocampal neuronal cells were co-transfected with wild-type (WT) or mutant (mut) sPLA2-IIA 3′-UTR-luciferase reporter constructs and miR-122 mimics or NC, respectively, the relative luciferase activities were measured. **(B,C)** Primary human and mouse astrocytes, and HT22 mouse hippocampal neuronal cells were transfected with miR-122 mimics and NC, **(B)** the expression of sPLA2-IIA were measured by western blot, **(C)** the expression of sPLA2-IIA were measured by qRT-PCR. Each point represents the mean ± SD. Data show a representative of three independent experiments. *p < 0.05, **p < 0.01.

Previous studies have suggested that human group sPLA2-IIA induces neuronal cell death via apoptosis. Exploratory Gene Association Networks have elucidated sPLA2-IIA as a prospective target of miR-122. To ascertain the direct interaction between miR-122 and the 3′-UTR of sPLA2-IIA, we performed the dual-luciferase reporter assay as a further investigation. The results demonstrated that miR-122 overexpression inhibited luciferase activity of the reporter gene in the WT construct, while it had no effect on the sPLA2-IIA-MUT construct (Figure 4A). To explore the potential impact of miR-122 on sPLA2-IIA regulation, we further examined sPLA2-IIA expression in cells following transfection with either miR-122 or NC mimics. Our findings demonstrate that upregulation of miR-122 markedly reduced the sPLA2-IIA expression at both mRNA and protein levels in astrocytes, as well as HT22 cells (Figures 4B,C), suggesting that sPLA2-IIA might be a direct target of miR-122 in astrocytes.

## Discussion

IS ranks among the leading causes of adult mortality globally. Despite significant breakthroughs in diagnostic technologies and novel clinical therapies for IS (24–26), the prognosis remains suboptimal, and the mechanisms underlying IS remain a subject of controversy. MiRNAs are emerging as key molecular mediators in IS and are considered potential diagnostic and therapeutic agents for this condition (27). Earlier research has indicated a significant reduction in miR-122 levels in animal models of ischemic cerebral reperfusion and the blood of patients with acute stroke (18). Our previous study demonstrated that treatment with miR-122-5p mimics reduces the volume of cerebral infarction in rats underwent MCAO/R. Furthermore, it validated that miR-122-5p reduces iNOS expression in leukocytes and BMVECs, suggesting a cerebroprotective role for miR-122 in IR injury following IS (21, 28). We have indicated that overexpression of miR-122 reverses cell apoptosis and cleaved caspase-3 level resulting from OGD/R in primary astrocytes and HT22 cells. Furthermore, our research has shown that overexpression of miR-122 results in decreased levels of IL-6, TNF-α, and ROS. Moreover, we found that overexpression of miR-122 leads to its direct binding to sPLA2-IIA 3′-UTR and the inhibition of sPLA2-IIA expression.

The PLA2 superfamily of enzymes comprises six subfamilies: cytosolic PLA2s, calcium-independent PLA2s, sPLA2s, lysosomal PLA2s, PAF acetylhydrolases, and adipose-specific PLA2s. These subfamilies play crucial roles in maintaining cellular membrane homeostasis under physiological conditions. Among them, sPLA2 play a pivotal role in inflammation-related diseases. sPLA2 hydrolyzes oxidized phospholipids in LDL cholesterol, generating oxidized free fatty acids. These fatty acids are precursors to the production of inflammatory substances such as AA, which is assumed to be associated with neuronal apoptosis (29). Also damages the vascular endothelium. Therefore, sPLA2 has been suggested as a vasculature-specific marker of inflammation (22). In the early stage after MCAO/R, a notable increase in sPLA2-IIA mRNA expression has been observed in the cerebral cortex of rats, which is attributed to the activation of astrocytes (30). However, Wang’s study has also indicated that the autocrine levels of sPLA2-IIA possibly has a protective effect in maintaining the integrity of BMVECs and reducing the increased permeability induced by lipopolysaccharide (31). These findings suggest a close association between sPLA2-IIA and IS. Importantly, a previous study has demonstrated that in the MCAO/R rat model, the levels of sPLA2-IIA in the penumbra did not increase in the early stage of ischemia, but an elevation in sPLA2-IIA levels was observed in the later stage (30). Therefore, sPLA2-IIA inhibitors, which can halt the reversible pro-apoptotic state in the penumbra, hold promise for reducing ischemic damage and facilitating the successful treatment of stroke.

At present, the standard recommended treatment for IS involves intravenous thrombolysis or interventional methods aimed at achieving vascular recanalization (32–34). These treatments are bound by strict time window requirements and carry the risk of secondary cerebral hemorrhage and reperfusion injury (24, 35). Owing to the BBB, antioxidant drugs are unable to reach the ischemic injury site, thereby limiting their biological impact. Consequently, the efficacy of existing drug therapies is limited. MiRNAs are implicated in various human disorders. Possessing high stability in human fluids, miRNAs are promising biomarkers for disease diagnosis and prognosis. Additionally, miRNA-based therapeutics hold the potential to revolutionize the treatment of diverse human pathologies (14, 36, 37). Emerging evidence suggests that miRNA expression is upregulated during stroke and is crucial in regulating the prognosis of stroke patients (21, 28). Therefore, therapeutic and diagnostic methods in stroke management can potentially gain valuable insights from the stroke–miRNA system. Previous studies have demonstrated a significant reduction in miR-122 levels in both animal models of ischemic cerebral reperfusion and the blood of patients with acute stroke (17, 19). Our previous study demonstrated that treatment with miR-122-5p mimics effectively reduces the volume of cerebral infarction in rats subjected to MCAO/R (20, 21). In line with these important observations, we validated that miR-122 mitigates astrocyte apoptosis following IS. Moreover, we elucidated sPLA2-IIA as a novel target gene of miR-122 and demonstrated that the upregulation of miR-122 markedly inhibits sPLA2-IIA expression, thereby suggesting a cerebroprotective role for the miR-122/sPLA2-IIA axis in IR injury following IS.

Emerging evidence suggests that astrocyte-mediated inflammatory responses play a vital role in IS and have emerged as a prime target for novel therapies for stroke (2, 20, 38). Astrocytes, the predominant cell type in the brain in terms of both quantity and volume, are responsible for regulating neuronal cell development and maintaining extracellular environmental homeostasis. In pathological conditions, including IS, astrocytes become activated and release adhesion molecules, chemokines, and other inflammatory factors into the affected areas. This secretion directly or indirectly contributes to the exacerbation of brain damage, neuronal dysfunction, microglial activation, and the recruitment of peripheral immune cells. Previous studies have demonstrated that sPLA2-IIA serves as an inflammatory mediator, stimulating the generation of pro-inflammatory cytokines and chemokines. Our results further validate that overexpression of miR-122 significantly decreases the expression of TNF-α and IL-6 in astrocytes. This finding suggests a cerebroprotective role for miR-122 in IS. However, additional investigations are necessary to explore the impact of miR-122 on post-ischemic inflammation, long-term survival, and functional recovery outcomes in an animal model of MCAO/R.

ROS have traditionally been considered as harmful byproducts of mitochondrial metabolic activities and a primary injury factor contributing to macromolecular damage in various inflammatory-related diseases, including IS. ROS production has been demonstrated to remarkably increase during IR injury (15). Although the sources of these ROS remain a subject of debate, IR injury arises from the interruption and subsequent restoration of blood supply to an organ, resulting in an increase in mitochondrial ROS production. Moreover, the aberrant accumulation of ROS can induce mitochondrial dysfunction, promote the generation of pro-apoptotic proteins, and trigger apoptosis, underscoring the pivotal role of ROS in reperfusion damage (39). Based on this research, we have revealed that miR-122 overexpression effectively reduces ROS production in astrocytes. Moreover, our findings demonstrate that the upregulation of miR-122 also decreases both the mRNA and protein expression of iNOS in astrocytes. Previous studies have suggested that iNOS is induced after 12 h, in the later phases of cerebral ischemia. The neurotoxic nitric oxide synthesized by iNOS has been implicated in impeding the delayed recovery from neuronal damage in the brain (40). Therefore, miR-122 may be involved in mitigating oxidative stress through multiple mechanisms, thereby exerting cerebroprotective effects in IS.

In summary, this particular research demonstrated that overexpression of miR-122 diminishes astrocyte apoptosis following an IS. Furthermore, we have identified sPLA2-IIA as a novel target gene of miR-122, with miR-122 directly binding to the 3′-UTR of sPLA2-IIA, leading to the inhibition of sPLA2-IIA expression. This inhibition, in turn, mitigates post-ischemic inflammation and reduces the production of ROS. Our research suggests an innovative function for miR-122 in IS, as well as highlights its potential utility in intervention strategies for IS. Nevertheless, further study utilizing animal models is warranted to contribute to a thorough comprehension of the vital effects of miR-122 in IS, particularly in relation to post-ischemic inflammation, long-term survival, and functional recovery outcomes.

## Data availability statement

The original contributions presented in the study are included in the article/supplementary material, further inquiries can be directed to the corresponding authors.

## Author contributions

YY: Data curation, Formal analysis, Investigation, Visualization, Writing – original draft. PL: Data curation, Formal analysis, Investigation, Visualization, Writing – original draft. MC: Data curation, Formal analysis, Investigation, Visualization, Writing – original draft. WZ: Formal analysis, Visualization, Writing – original draft. TZ: Conceptualization, Formal analysis, Writing – original draft. LM: Conceptualization, Methodology, Writing – review & editing. HL: Data curation, Formal analysis, Validation, Writing – review & editing, Writing – original draft. BL: Conceptualization, Funding acquisition, Methodology, Project administration, Resources, Supervision, Writing – review & editing.
